# Application of Digital Twin Technology to Enhance Chronic Diseases Management: A Systematic Review

**DOI:** 10.1155/ijta/2299762

**Published:** 2026-06-10

**Authors:** Amirhossein Zarei, Marsa Gholamzadeh, Fatemeh Asadi

**Affiliations:** ^1^ Health Information Technology, Health Information Management and Medical Informatics Department, School of Allied Medical Sciences, Tehran University of Medical Sciences, Tehran, Iran, tums.ac.ir; ^2^ Thoracic Research Center, Imam Khomeini Hospital Complex, Tehran University of Medical Sciences, Tehran, Iran, tums.ac.ir; ^3^ Health Information Management and Medical Informatics Department, School of Allied Medical Sciences, Tehran University of Medical Sciences, Tehran, Iran, tums.ac.ir; ^4^ Health Information Management Department, School of Health Management and Information Sciences, Kerman University of Medical Sciences, Kerman, Iran, kmu.ac.ir

**Keywords:** artificial intelligence, chronic disease management, digital twins, healthcare technology, personalized medicine, systematic review

## Abstract

**Background:**

Given that chronic diseases account for a considerable proportion of preventable deaths globally, the adoption of innovative technologies for disease management and prevention is crucial. Digital twins (DTs), representing one of the most advanced technological solutions, enable real‐time simulation and monitoring of chronic disease progression, facilitating personalized treatment strategies and early intervention. This systematic review examines current research on DT applications in chronic disease management to evaluate their potential impact.

**Methods:**

A systematic search was conducted in four databases including PubMed, Scopus, Web of Science, and IEEE from inception to the date of the last search. The research question was formulated using PICO framework. Next, all articles were screened following Preferred Reporting Items for Systematic Reviews and Meta‐Analyses (PRISMA) guidelines to select eligible articles based on inclusion criteria. The extracted information was analyzed to determine the main applications, domains, and employed technologies using quantitative and qualitative techniques.

**Results:**

Out of 298 citations, 20 studies met our inclusion criteria after duplicate removal and screening. Most studies (45%, *n* = 10) were published between 2023 and 2024, indicating an increasing trend in this area. Geographically, the United States contributed the most studies (25%, *n* = 5), followed by Switzerland (15%, *n* = 3). Our analysis revealed that primary applications of DT in chronic disease management included medical training and education (65%, *n* = 13), personalized medicine and patient care (45%, *n* = 9), and drug discovery and clinical trials (35%, *n* = 7). Target groups comprised clinicians (42.11%), patients (31.58%), and medical students (15.79%). Key enabling technologies in this subject were data analytics (65%), artificial intelligence and machine learning (60%), computational physiological modeling (30%), and IoT sensors (25%).

**Conclusions:**

Our findings demonstrate that DT technology has evolved from theoretical models to integrated clinical applications, with the potential to revolutionize healthcare through personalized medicine, continuous monitoring, and AI‐driven decision support.

## 1. Introduction

Chronic diseases—such as diabetes, cardiovascular disorders, respiratory diseases, and kidney diseases—are among the most significant health challenges today. According to the World Health Organization (WHO), they account for the overwhelming percentage of preventable deaths and disabilities, making them a critical issue in healthcare worldwide [1–3]. Despite technological advancements, traditional approaches usually fail to address the dynamic and diverse nature of chronic diseases, leading to delayed interventions and increased healthcare costs [4, 5]. To achieve effective, patient‐centered outcomes, there is a pressing need for innovative solutions leveraging new technologies.

One of the most innovative technologies today is the digital twins, a concept first developed in industrial engineering and now transforming healthcare [6, 7]. A DT is a digital copy or virtual representation of a physical object or system designed to simulate the behavior, function, or process of a real concept to gain a better understanding of how it functions in real time by adding new data [8, 9]. This concept has been increasingly adopted across health and medical sciences [10, 11], demonstrating strong potential to transform clinical practice by enabling real‐time patient monitoring, prediction of disease progression, and personalization of treatment strategies [12, 13]. The integration of DT technology in healthcare represents a paradigm shift towards a personalized medicine approach [14]. The importance of DTs in healthcare lies in their ability to create virtual versions of patients, which can simulate real‐time health data and predict disease progression in complex diseases [15, 16]. This innovative approach allows treatment plans to be tailored to the patient’s needs, ultimately improving disease management outcomes and reducing healthcare costs [17].

DT technology offers significant potential for real‐time monitoring and analysis, which can substantially enhance decision‐making processes in healthcare, particularly in chronic disease management [18]. Understanding the applications and effectiveness of DT‐based approaches is crucial for both healthcare practitioners and policymakers. This systematic review aims to synthesize existing research on DT applications in chronic disease management, critically examining the potential benefits, inherent challenges, and promising future research directions. Through a comprehensive literature analysis, the review seeks to provide nuanced insights and evidence‐based recommendations for the strategic implementation of DT solutions in healthcare settings and its potential to revolutionize precision medicine for chronic diseases.

Although DTs share similarities with conventional simulation models, they differ in several important ways that guided our inclusion criteria [7, 9, 11, 13]. Traditional simulation models are often implemented as standalone, predefined representations and are not intrinsically linked to continuously updated data streams from a specific real‐world patient or physiological system [9, 11, 13]. In contrast, a DT represents a dynamic, and data‐driven model of an individual patient or physiological system [7, 11–13]. DTs incorporate real‐time or longitudinal data, adapt their internal state based on new inputs, and generate personalized predictions [10–13]. Therefore, we excluded studies that used simulations without an explicit mechanism for continuous updating and/or patient‐specific adaptation, and included studies that incorporated adaptive and patient‐specific modeling components [7, 9, 11, 13].

## 2. Methodology

This systematic review was conducted in accordance with the Preferred Reporting Items for Systematic Reviews and Meta‐Analyses (PRISMA) guidelines. A comprehensive literature search was performed across four electronic databases—MEDLINE via PubMed, Scopus, Web of Science (WoS), and IEEE—without any time restrictions, using relevant keywords and Medical Subject Headings (MeSH) terms [19, 20]. The detailed search strategies for each database are provided in Appendix A (Table A1).

To ensure the rigor of the screening process and accurate reference management, EndNote (version 21) and the web‐based Rayyan software were used. The review employed the PICO (Population, Intervention, Context) framework to structure the search strategy. The population included patients with chronic diseases. The intervention of interest comprised DT‐based interventions or programs aimed at enhancing chronic disease care. The context focused on the application of DT technologies within healthcare settings.

Based on the research question, studies were considered eligible if they reported the use of DT technology to improve patient care in chronic disease management and presented quantitative and/or qualitative outcomes in the form of peer‐reviewed scientific publications. This review specifically focused on studies aimed at improving patient care outcomes, with particular emphasis on those promoting active patient engagement in care processes.

### 2.1. Inclusion and Exclusion Criteria

Before the systematic screening process began, the authors predefined the inclusion and exclusion criteria for this descriptive, exploratory review in alignment with the study’s research objectives and questions. Eligible studies included DT‐based interventions covering any phase of chronic disease care, including comprehensive care and remote follow‐up approaches; studies with a clear focus on chronic diseases; and peer‐reviewed publications available in English. Studies were excluded if they were editorial letters, commentaries, perspectives, brief reports, theses or dissertations, book chapters or reviews, short communications, abstract‐only publications, unpublished materials, non‐English publications, duplicate records, or existing systematic reviews and narrative literature reviews.

During screening, a substantial number of records were excluded under the category “Wrong subject.” In this review, “Wrong subject” referred to studies in which the term DT was used outside the context of healthcare or chronic disease management. Specifically, these studies applied DT concepts to nonclinical domains such as manufacturing systems, industrial automation, robotics, mechanical engineering, predictive maintenance, smart factory design, or medical device production pipelines. Although such studies used DT terminology, they did not involve patients, physiological modeling, clinical data, or healthcare‐related decision support and were therefore outside the scope of this review.

Similarly, the category “Wrong publication type” was used for records that did not meet the requirement of being full‐text, peer‐reviewed scientific publications. These sources included editorials, commentaries, opinion pieces, magazine articles, abstracts without an accompanying full paper, posters, workshop summaries, non–peer‐reviewed conference notes, and technical white papers. To ensure methodological rigor and to adhere to PRISMA recommendations regarding prespecified eligibility criteria and transparent reporting, only full‐text, peer‐reviewed journal articles and conference papers were included.

### 2.2. Screening Process

Study screening was performed in accordance with the PRISMA guidelines. After removing duplicate records from the database searches, two reviewers independently screened titles and abstracts to exclude clearly irrelevant studies. Full texts of potentially eligible articles were then assessed against the predefined inclusion and exclusion criteria. Data were extracted using a standardized form capturing study objectives, methodological characteristics, and key outcomes. Any disagreements regarding study eligibility were resolved through discussion and, when necessary, adjudication by a third reviewer. This process ensured a transparent and reproducible study selection procedure.

### 2.3. Quality Assessment

Methodological quality was appraised using the Mixed Methods Appraisal Tool (MMAT; 2018 version) [21]. For each included empirical study, we first applied the two MMAT screening questions (S1–S2) and then categorized the study according to its design (qualitative, randomized controlled trial, nonrandomized, quantitative descriptive, or mixed methods) [21]. Included empirical studies were subsequently assessed against the five design‐specific MMAT criteria and rated as “Yes,” “No,” or “Cannot tell” [21]. Nonempirical papers (e.g., conceptual architectures, technical frameworks, or protocol papers without empirical data) were not appraised beyond the screening stage because the MMAT is intended for empirical primary research and is not applicable to theoretical or review papers [21]. In accordance with MMAT guidance, we did not calculate an overall numeric quality score; instead, we reported criterion‐level ratings for each appraised study [21]. A summary of the MMAT appraisal is presented in Appendix B, Table B1.

## 3. Results

**Figure 1:**
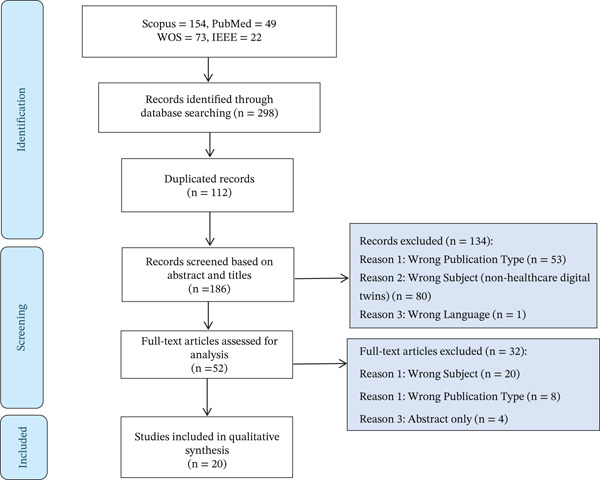
The PRISMA diagram for screening process.

The database searches identified 298 records. After removing 112 duplicates, 186 records remained for title and abstract screening. Of these, 52 full‐text articles were assessed for eligibility, and 20 studies met the inclusion criteria and were included in the qualitative synthesis. The study selection process, including the reasons for exclusion and the corresponding numbers, is presented in Figure 1.

### 3.1. General Characteristics

An analysis of the included studies showed that prototype development and evaluation and computational modeling and simulation were the most frequently addressed themes, each reported in nine studies (40%). Conceptual framework development was less commonly reported (10%, *n* = 2), while observational studies and pharmacodynamics modeling were the least represented categories, each accounting for 5% (*n* = 1) of the included literature. It should be noted that these thematic categories were not mutually exclusive, and individual studies could be classified under more than one category.

The included studies were published between 2008 and 2024, demonstrating a clear increase in research activity in recent years. Notably, nearly half of the studies (45%, *n* = 10) were published during the period 2023–2024.

**Figure 2:**
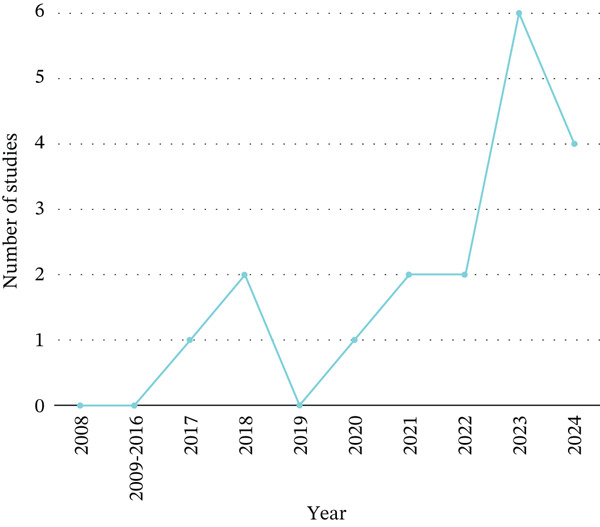
Timeline of published DT studies in chronic disease management (2008–2024).

Figure 2 illustrates the temporal trend in DT publications related to chronic disease management. Publication volume remained limited until 2018, followed by steady growth and a pronounced increase during 2021–2023, indicating rising research attention in this area.

**Figure 3:**
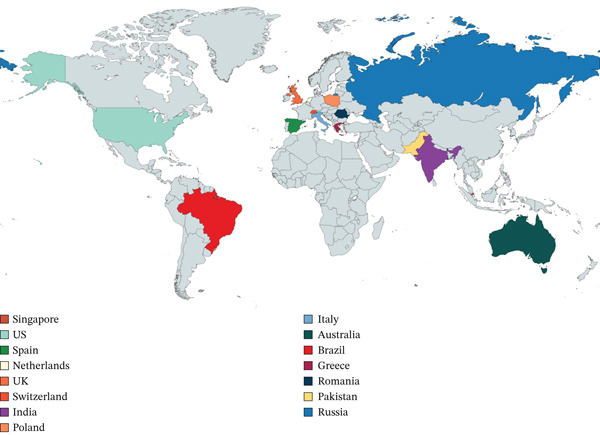
The geographical distribution of the included articles.

An analysis of the geographical distribution of the included studies showed contributions from 13 countries (Figure 3). The United States contributed the largest number of studies (*n* = 5, 25%), followed by Switzerland (*n* = 3, 15%) and the United Kingdom (*n* = 2, 10%). This distribution suggests broad international research activity on the application of DT technologies across diverse healthcare settings.

Figure 3 shows the geographical distribution of the included studies, indicating that published research on DT applications in chronic disease management is uneven across countries. This pattern may reflect differences in digital health infrastructure, research capacity, and access to enabling technologies that support DT development and evaluation. In particular, publications were concentrated in a limited number of countries, whereas few or no eligible studies were identified from several regions, suggesting potential barriers to DT‐related research and implementation in those settings. These findings highlight the value of broader international collaboration and capacity‐building initiatives to support more equitable development and adoption of DT technologies worldwide.

Sample sizes varied substantially across the included studies, ranging from 8 to 7081 participants or cases. Virtual cohorts were frequently used, with some studies simulating large populations (up to 3000 virtual patients [VPs]). Studies involving real participants ranged from small samples (8–40 participants) to large cohorts (more than 1000 participants). Six studies either did not report a sample size or were conceptual/framework papers for which a sample size was not applicable. Among studies reporting sample sizes for real participants, the mean sample size was 894.5 ± 2,156.8 (median, 40; IQR, 20–259). The general characteristics of the included studies are summarized in Table 1.

**Table 1:** The general characteristics of included articles.

#	Author names	Year	Type of study	Clinical discipline	Application analysis	Aim (develop/evaluate)	Sample size (*n*)	Key outcome/metric (reported)	Other enabling technologies	Simulation/DT core approach	Validation level
1	Surian N et al. [22]	2024	Computational modeling and simulation	Nephrology	Predictive analytics and risk management	Develop a tool	7081	This holistic methodology provides insights into patients’ health states and CKD progression rates based on GMF metabolic profile difference.	Logistic regression analysis	AI algorithms	Retrospective/validation (*n* ≥ 50)
2	Zhang Y et al. [23]	2024	Conceptual framework development	Endocrinology	Personalized medicine and patient care	Framework development	1356	Proposed a framework for digital twin development in type 2 diabetes	Artificial intelligence, machine learning, data analytics	Not specified	Conceptual/architecture
3	Mancini, E et al. [24]	2018	Computational modeling and simulation	Infectious Disease	Drug discovery and clinical trials	Model development	500	The in‐silico approach provided insights into the dynamics of temporary HIV treatment and helped explain the contradictory results	Mathematical modeling, data analysis	Silico approach and agent‐based modeling and simulation	Retrospective/validation (*n* ≥ 50)
4	Chakshu, N K and Nithiarasu, P [25]	2022	Computational modeling and simulation	Pulmonary Medicine	Healthcare operations and management—predictive analytics and risk management	Model development	493	A prototype AI‐based digital twin system for prioritizing pneumonia patient treatment was developed and evaluated	Machine learning, data analytics	Not specified	Retrospective/validation (*n* ≥ 50)
5	Bahrami F et al. [26]	2023	Pharmacodynamics modeling	Pharmacology	Personalized medicine and patient care	Model development and evaluation	3000	A prototype individualized digital twin for transdermal fentanyl therapy in chronic pain management was developed and evaluated	Pharmacokinetic/pharmacodynamic modeling, machine learning, data analytics	Digital twin simulation	Retrospective/validation (*n* ≥ 50)
6	Sai S et al. [27]	2024	Conceptual framework development	General Medicine	Personalized medicine and patient care—remote patient monitoring	Framework development	Not mentioned	A conceptual framework integrating AI‐powered digital twins and NFT‐based patient monitoring for chronic disease management was proposed	Artificial intelligence, machine learning, blockchain, Internet of Things	Not specified	Conceptual/architecture
7	Li G et al. [28]	2024	Prototype development and evaluation	Oncology	Personalized medicine and patient care	The article presents a novel application of digital twin technology to improve the management of chronic graft‐versus‐host disease,	2042	A prototype digital twin system for chronic graft‐versus‐host disease patients was developed and evaluated	Machine learning, data analytics, predictive modeling	Trial accelerator digital twin platform	Retrospective/validation (*n* ≥ 50)
8	Semakova, A and Zvartau, N [29]	2018	Computational modeling and simulation	Medical Education	Drug discovery and clinical trials—predictive analytics and risk management	Develop a model for simulating patient populations	40606	A computational framework for identifying hypertensive patient profiles and simulating patient populations was developed and evaluated	Data mining, machine learning, statistical modeling	Agent‐based modeling and simulation	Retrospective/validation (*n* ≥ 50)
9	Ho F et al. [30]	2022	Computational modeling and simulation	Medical Education	Predictive analytics and risk management—drug discovery and clinical trials	Develop a framework	Not mentioned	A virtual imaging trial framework for evaluating airway quantification algorithms was developed and demonstrated	Image processing, machine learning, data analysis	Virtual imaging simulation	Simulation‐only/unclear
10	Sarp S et al. [31]	2023	Prototype development and evaluation	General Medicine	Remote patient monitoring—personalized medicine and patient care	Model development	Not mentioned	A prototype digital twin system for chronic wound management was developed and evaluated	Machine learning, data analytics, sensor technologies	Not specified	Simulation‐only/unclear
11	Gólczewski T et al. [32]	2017	Prototype development and evaluation	Pulmonary Medicine	Medical training and education—drug discovery and clinical trials	The aim of developing the virtual patient (VP) was to analyze the impact of physiological factors on pleural pressure (Pp) during therapeutic thoracentesis.	32	Identification of key physiological factors that influence pleural pressure during thoracentesis.	Mathematical modeling of the respiratory system, computer simulation of physiological pressure	The study uses a virtual patient (VP) to simulate the respiratory system.	Pilot/feasibility (*n* < 50)
12	Cukic M, et al. [33]	2024	Prototype development and evaluation	Oncology	Personalized medicine and patient care—drug discovery and clinical trials	To validate a physics‐based digital twin (DT) for predicting individual fentanyl plasma concentrations. To develop a tool for personalized medicine that uses a combination of physics‐based models and patient‐specific data. To create a tool that can provide improved initial dosing and constant data‐based adaptation of further dosing.	19	Individual fentanyl Cmax concentrations (primary endpoint).	The study will use therapeutic drug monitoring to validate DT predictions.	This study uses a physics‐based digital twin (DT) that includes:	Pilot/feasibility (*n* < 50)
13	Coto‐Segura P et al. [34]	2023	Prototype development and evaluation	Rheumatology	Drug discovery and clinical trials	Develop and evaluation a model to assess the performance of our model in generating a vPop and defining CZP response variability based on patient profiles.	Not mentioned	A validated quantitative systems pharmacology model for certolizumab pegol treatment in psoriasis was developed.	Pharmacokinetic/pharmacodynamic modeling, machine learning simulation technology.	TPMS technology	Simulation‐only/unclear
14	Bahrami F, et al. [35]	2022	Computational modeling and simulation	General Medicine	Personalized medicine and patient care	The aim is to develop a physics‐based digital twin of a patient for fentanyl delivery, which takes into account drug diffusion through the skin, PK, and PD models, and patient feedback.	20	The main outcomes include the development of a physics‐based digital twin for personalized fentanyl therapy.	To simulate drug diffusion through the skin, Physiologically based…	This study utilizes physics‐based simulations within a digital twin framework, combining …	Pilot/feasibility (*n* < 50)
15	Koulas I, et al. [36]	2021	Prototype development and evaluation	Rheumatology	Medical training and education	The aim was to develop and evaluate a mobile application that uses virtual patient scenarios to educate patients with chronic pain and improve their self‐management skills	20	The study demonstrates that a mobile application using virtual patients can be a valuable tool for patient education	Progressive web application technology. xAPI learning	The study uses virtual patients (VPs), which are interactive, computer‐based simulations	Pilot/feasibility (*n* < 50)
16	Larkin A et al. [37]	2020	Prototype development and evaluation	Medical Education	Medical training and education	The aim was to evaluate whether a virtual patient simulation (VPS) can improve cardiologists’ clinical decisions related to the identification and management of hyperkalemia	122	The study concluded that virtual patient simulation is an effective way to improve evidence‐based clinical decisions related to patient.	The intervention provided tailored clinical guidance (CG) based on current evidence.	The study uses virtual patient simulations (VPS) where learners can order lab tests.	Retrospective/validation (*n* ≥ 50)
17	Maleki A et al. [38]	2023	Computational modeling and simulation	Neurology	Drug discovery and clinical trials—predictive analytics and risk management	The aim is to validate and expand the Universal Immune System Simulator (UISS‐MS) as a digital twin solution for multiple sclerosis. The UISS‐MS platform is designed to simulate the human immune system and the effects of treatments for MS. The study aims to validate the model’s ability to simulate the effects of cladribine and ocrelizumab.	3000	The main outcomes of the study include the following: ○ Successful validation of UISS‐MS for cladribine and ocrelizumab treatments.	The study references the use of clinical trial data.	The study utilizes the Universal Immune System Simulator (UISS), an agent‐based model.	Retrospective/validation (*n* ≥ 50)
18	Park C et al. [39]	2021	Observational study	Geriatric Medicine	Remote patient monitoring	The aim of this study is to identify the optimal digital biomarkers of physical frailty that can be measured using a pendant sensor during activities of daily living. The study uses machine learning to determine the least number of sensor‐derived features needed to identify physical frailty and its phenotypes	259	The main outcomes of the study include the following: ○ Identification of four optimal sensor‐derived features (% of standing, % of walking).	The study uses machine learning, specifically logistic regression modeling.	This study did not employ simulation technology. It is based on empirical data collected …	Retrospective/validation (*n* ≥ 50)
19	Khan S et al. [40]	2022	Computational modeling and simulation	General Medicine	Remote patient monitoring	The aim of this study is to evaluate the performance and data collection capability of unobtrusive microwave sensors in a care‐home environment to provide a foundation for creating a DT model with realistic patient data. The study focuses on using microwave‐sensing technology, unobtrusively, for data collection in a static care‐home model	1002	The main outcomes of the study include the following: ○ A detailed analysis of an unobtrusive microwave sensor’s performance in a simulated care‐home environment.	The study utilizes microwave‐sensing technology, 3D modeling in SolidWorks, and simulation.	This study uses 3D modeling and simulation software to create a virtual care‐home environment.	Retrospective/validation (*n* ≥ 50)
20	Khan Sa., et al. [41]	2024	Prototype development and evaluation	General Medicine	Remote patient monitoring	To improve the efficiency and reduce the cognitive load on medical professionals by enabling autonomous navigation of the physical twin (PT) robot in a remote patient monitoring system using a digital twin (DT) framework.	Not mentioned	A real‐time RPM system with autonomous navigation, obstacle avoidance, and data transmission capabilities. The system showed high navigation.	Digital twins, Internet of Robotic Things (IoRT), virtual reality (VR), Bluetooth, NRF24L…	The study uses a virtual environment (VE) for visualization and simulation.	Pilot/feasibility (*n* < 50)

### 3.2. Clinical Aspects

Across clinical disciplines, the included studies spanned general medicine (*n* = 5, 23.81%), medical education (*n* = 3, 14.29%), pulmonary medicine (*n* = 2, 9.52%), pharmacology (*n* = 2, 9.52%), and oncology (*n* = 2, 9.52%). Additional specialties—including endocrinology, geriatric medicine, nephrology, and neurology—were each represented by one study (*n* = 1, 4.76%).

In terms of clinical conditions, chronic pain was the most frequently addressed topic (*n* = 3, 14.29%). Other conditions included COPD, cancer, pneumonia, psoriasis, and several additional disease areas. Overall, DT applications were reported across a broad range of clinical domains, indicating wide applicability within healthcare.

### 3.3. Target Groups

Across the included studies, target populations were grouped into five categories: clinicians (42.11%) [23, 26, 35–37, 41], patients (31.58%) [27, 34, 38–40], medical students (15.79%) [29, 37, 41], and studies targeting both patients and clinicians (10.53%) [34]. One study targeted multiple stakeholder groups. Because some studies addressed more than one target population, these categories were not mutually exclusive.

Among healthcare professionals, physicians were the most frequently targeted group, including specialists such as intensivists, transplant physicians, cardiologists, and radiologists. The clinician category also encompassed clinical researchers, health policymakers, and other members of the scientific community, particularly in studies proposing conceptual frameworks or examining DT applications at the health‐system level. Overall, the presence of studies with multiple target populations reflects the integrated nature of DT applications in chronic disease care.

### 3.4. Different Applications of Digital Twin in Chronic Disease Management

**Figure 4:**
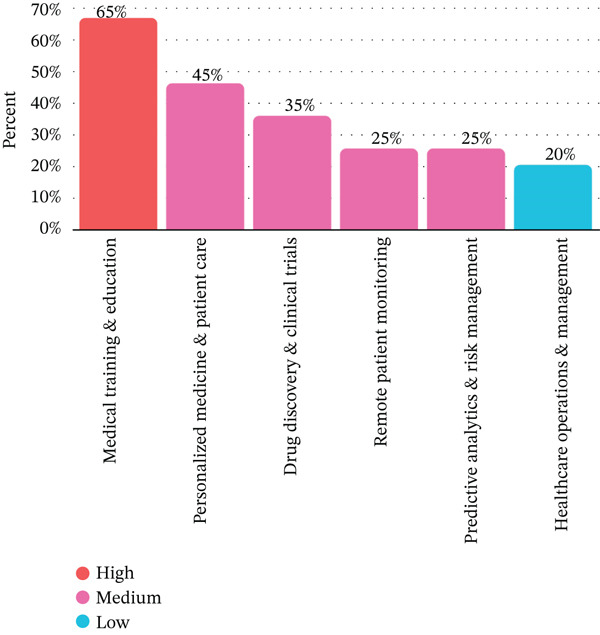
Different applications of DT in chronic disease management.

An analysis of the included studies showed that DT applications in chronic disease management could be grouped into several application areas. Because many studies addressed more than one application area, these categories were not mutually exclusive. Medical training and education was the most frequently reported application (65%, *n* = 13), followed by personalized medicine and patient care (45%, *n* = 9). Applications related to drug discovery and clinical trials accounted for 35% (*n* = 7). Remote patient monitoring (RPM) and predictive analytics/risk management were each reported in 25% of studies (*n* = 6 each). Healthcare operations and management was the least frequently reported application area (20%, *n* = 5). Overall, these findings indicate a broad and growing range of DT applications, with a particular emphasis on education and patient‐centered care. To facilitate interpretation, application areas were further classified based on their frequency across the included studies: “high” (> 50% of studies), “medium” (25%–50%), and “low” (< 25%). The distribution of application domains and their relative frequencies are presented in Figure 4.

**Figure 5:**
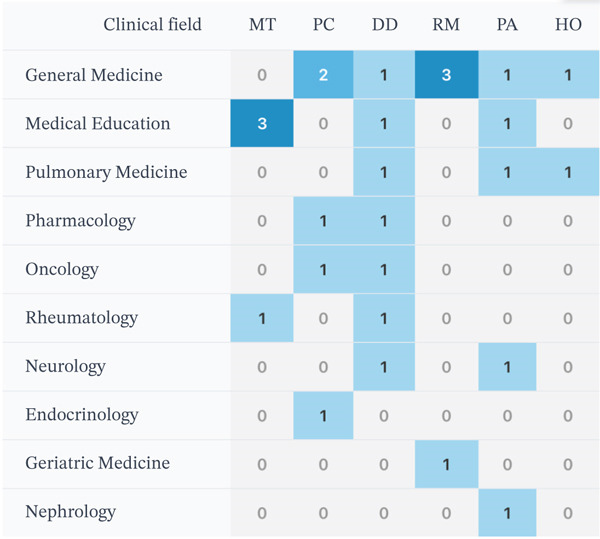
Heatmap of the relationship between clinical disciplines and DT application types (MT, medical training; PC, personalized care; DD, drug discovery; RM, remote monitoring; PA, predictive analytics; HO, healthcare operations).

Figure 5 provides a visual overview of the distribution of DT application types across clinical disciplines. Medical training and education, personalized care, and drug discovery were observed across multiple clinical fields, whereas remote monitoring and predictive analytics were primarily concentrated in general medicine, pulmonary medicine, and geriatric care. Overall, the heat map indicates variability in how DT applications are represented across clinical specialties. Application domains in terms of various disciplines are represented in Appendix B, Table B1.

#### 3.4.1. Medical Training and Education

Several included studies reported the use of DT‐enabled approaches for medical training and education through a range of methodologies. VP scenarios were commonly used to support clinicians in practicing diagnostic reasoning and clinical decision‐making in simulated settings [36, 37]. The study by Koulas et al. [36] described a mobile, scenario‐based learning approach for chronic pain management; although the study did not explicitly refer to DTs, it employed personalized simulated patient scenarios with interactive feedback that are conceptually related to DT‐informed training models. In addition, DT‐based educational platforms have been used to simulate realistic medical procedures, allowing trainees to practice complex interventions in a safe and controlled virtual environment (VE) [22].

Across studies in the medical training domain, DT‐aligned virtual platforms were associated with short‐term improvements in performance‐related outcomes. Larkin et al. reported post‐intervention improvements in clinical decision choices following a VP simulation–based continuing medical education (CME) activity [37]. Ho et al. reported improved airway quantification accuracy within a virtual imaging trial framework using VP models and ground‐truth comparisons [30]. Collectively, these findings suggest that DT‐enhanced training environments may support gains in technical precision and learning performance. However, the available evidence is largely limited to short‐term and/or technical performance endpoints, with no assessment of retention, real‐world transfer, or patient‐level impact [30, 37].

#### 3.4.2. Personalized Medicine and Patient Care

The included studies indicate that DTs are increasingly being used to support the development of personalized care strategies based on individual patient data [23, 26, 28]. For example, DT‐based approaches described by Bahrami et al. [26] and Li et al. [28] applied disease progression simulation and treatment‐response modeling to optimize chronic disease management by evaluating the potential effects of alternative therapeutic strategies. These applications reflect a transition from generalized treatment approaches toward more patient‐specific and data‐driven care models [23, 26]. Other studies reported that DT‐based models may improve the prediction of patients’ responses to specific treatments, with the potential to enhance clinical decision‐making and reduce the risk of adverse effects [26, 28].

Across the reviewed literature, DT applications in personalized care demonstrated promising technical fidelity in reproducing patient‐specific trajectories and physiological responses within model validation settings [22, 26, 35]. Studies by Surian et al. and Bahrami et al. reported that DT‐aligned computational and pharmacokinetic/pharmacodynamic (PK/PD) modeling approaches were able to approximate individualized disease progression patterns and drug response dynamics with favorable internal validation performance [22, 26, 35]. These patient‐specific models enabled individualized predictions beyond static, population‐average parameterizations and supported personalized forecasting under model‐specific assumptions [22, 26, 35]. However, despite demonstrated technical performance, none of the included studies assessed whether such personalized predictions translated into improved clinical outcomes, reduced hospitalizations, or enhanced patient safety in real‐world clinical settings [22, 26, 35].

Overall, the current evidence supports DT‐based approaches as technically feasible and internally validated tools for personalized care, while highlighting the absence of clinical validation through patient‐level outcome evaluation [10, 11, 13, 22, 26, 35].

#### 3.4.3. Drug Discovery and Clinical Trials

Several included studies examined the use of DTs o support optimization of drug treatment regimens, assess drug efficacy, and predict therapeutic outcomes [24, 29, 33, 34]. For example, Coto‐Segura et al. reported a computational modeling approach to treatment optimization in psoriasis, illustrating how DT‐aligned models can be used to simulate patient‐specific responses in a controlled modeling environment [34]. Related work also discussed the potential role of DT‐based or virtual cohorts as supportive components in clinical research (e.g., in trial design or comparative evaluation), thereby highlighting the relevance of DTs for drug development and evaluation [33]. DT‐enabled simulation of VP populations may complement traditional clinical trials by supporting early‐stage testing of novel drugs and therapeutic strategies and by reducing the resources required for exploratory evaluations [29]. Such approaches may be particularly valuable in rare diseases or highly personalized therapies, where recruiting sufficiently large patient cohorts is often challenging.

Studies applying DTs to drug discovery and dosing optimization reported that model‐generated outputs were calibrated and/or validated against clinical or trial‐derived data within the respective studies [24, 34, 38]. For example, Coto‐Segura et al. presented a quantitative systems pharmacology (QSP) model of certolizumab pegol (CZP) in moderate‐to‐severe psoriasis, describing an in silico approach to support treatment optimization and digital‐twin–like trial arms [34]. Similarly, Maleki et al. reported VP–based in silico modeling in multiple sclerosis (MS), incorporating treatment‐layer implementation and validation to demonstrate how simulation‐based platforms can be used to explore alternative therapeutic scenarios [38].

Collectively, these studies suggest that DT/QSP‐style platforms function primarily as hypothesis‐generating environments for evaluating alternative dosing strategies, treatment options, and therapeutic behavior under defined model assumptions [24, 34, 38]. However, the available evidence did not demonstrate downstream real‐world benefits, such as reduced drug development timelines, lower development costs, or improved patient safety attributable to DT implementation [34, 38]. Overall, most models remain at a proof‐of‐concept or simulation and validation stage, without prospective clinical deployment, thereby limiting conclusions regarding their real‐world impact [34, 38].

#### 3.4.4. RPM

RPM is a commonly reported application of DT technology in chronic disease care. In this review, several studies described DT‐enabled approaches for monitoring patients over time. For example, one study implemented a DT‐based monitoring system to track chronic wound healing progression [31], while another used DTs to screen and monitor physical frailty in older adults [39]. These RPM applications may support patient care by enabling real‐time monitoring of health status and facilitating more timely or responsive interventions [30]. In addition, DT‐enabled RPM may reduce reliance on in‐person consultations, which could be particularly beneficial for individuals in geographically remote areas or those with limited mobility [41].

DT‐enabled remote monitoring systems reported encouraging feasibility findings [31, 40]. For example, Sarp et al. described a DT‐based approach to track chronic wound healing progression, while Khan et al. reported an unobtrusive microwave‐sensing DT setup capable of detecting movement and vital sign–related changes without direct patient contact [31, 40]. These prototypes suggest that DTs may support continuous, low‐burden monitoring in out‐of‐clinic environments [31, 40]. However, evaluations were limited to small‐scale feasibility testing with minimal statistical validation, and neither study assessed downstream clinical outcomes such as improved healing rates, reduced complications, or earlier detection of deterioration [31, 40]. Overall, while feasibility evidence is available, evidence of clinical effectiveness remains insufficient.

#### 3.4.5. Predictive Analytics and Risk Management

Predictive analytics and risk management applications use DTs to forecast disease progression, assess risk factors, and support clinical decision‐making. For example, one study simulated populations of patients with hypertension to inform clinical trial design and related healthcare strategies [26]. More broadly, DT‐based predictive analytics may help clinicians manage care proactively by identifying individuals at higher risk and anticipating potential deterioration. In this review, Surian et al. reported that DT‐enabled modeling approaches can be used to identify at‐risk patients and predict potential health problems within the study setting [22].

#### 3.4.6. Healthcare Operations and Management

DT technology has been applied to support healthcare operations and hospital management. The included studies reported DT applications in areas such as intensive care resource allocation, optimization of hospital workflows, and broader facility management [33]. For instance, DT‐based simulation of patient flow patterns was used to estimate resource requirements and inform operational planning within hospital settings [25]. Overall, DT‐enabled operational models may help improve efficiency by supporting predictive modeling of patient demand and dynamic resource allocation [33]. However, evidence of downstream benefits—such as cost reduction, reduced waiting times, improved patient satisfaction, or improved clinical outcomes—remains limited in the current literature and is often not evaluated using patient‐level or system‐level outcome measures [25, 33].

DT applications for operational improvement—such as workflow optimization, equipment monitoring, and system‐level simulation—generally demonstrated technical feasibility, but real‐world operational gains were rarely quantified [25, 33, 40]. Although several models showed the potential to simulate patient flow, ventilation‐related behavior, or sensor‐placement performance, none reported implementation‐level outcomes measured in routine practice (e.g., cost reduction, improved resource allocation, or objectively measured operational efficiency) [25, 33, 40]. Overall, DT applications in healthcare operations remain promising but are largely conceptual or prototype‐based, underscoring the need for rigorous implementation and evaluation studies to establish real‐world effectiveness [25, 33].

**Figure 6:**
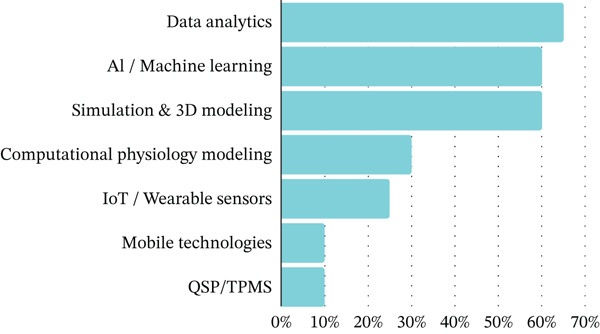
Frequency of enabling technologies used in DT studies for chronic disease management.

Figure 6 summarizes the frequency of enabling technologies reported across the included studies. Data analytics (65%), artificial intelligence (AI)/machine learning (ML) (60%), and simulation technologies (60%) were the most frequently reported components. Internet of Things (IoT) sensors (25%), computational physiological modeling (30%), mobile technologies (10%), and QSP/TPMS (approximately 10%) were reported less frequently.

### 3.5. Innovative Technologies Employed in DT

**Figure 7:**
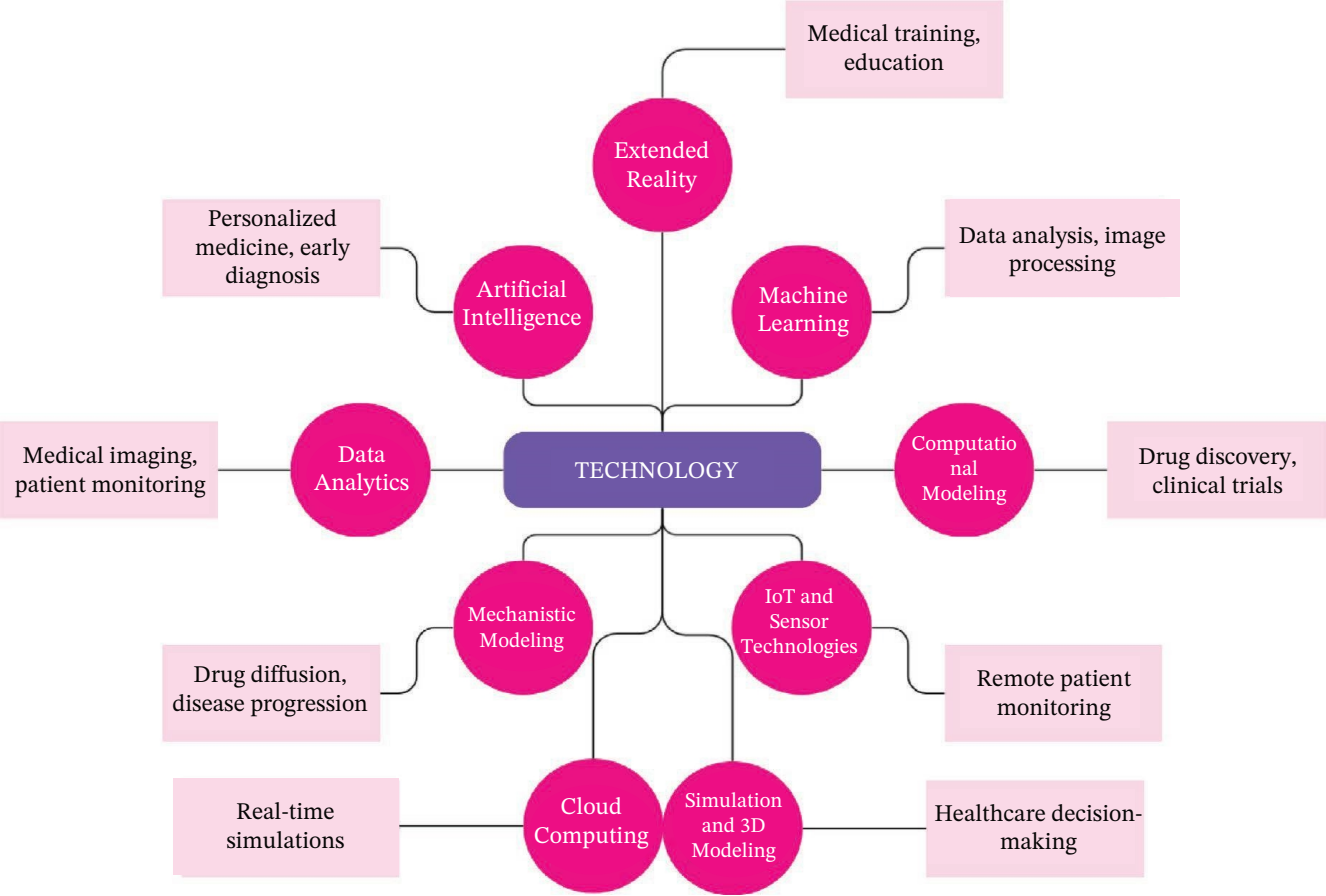
Different applications of DT in chronic disease management.

The successful implementation of DT technology in healthcare requires the integration of multiple complementary technologies. Our qualitative analysis of the included studies reveals that a wide range current DT systems commonly incorporate data analytics solutions (45%) [23, 25, 26, 29, 35–37, 39, 41], AI or ML techniques (60%) [25–27, 29, 30, 33–36, 39–41], computational physiological modeling (30%) [26, 34, 35, 37, 38], IoT services or wearable sensor (25%) [27, 33, 39–41], simulation and three‐dimensional (3D) modeling technologies (60%) [26, 29, 33–37, 41], and mobile technology (10%) [34, 38] to enhance the capabilities of DTs. Several studies employed multiple technologies simultaneously. These technologies and their associated subcategories are illustrated in Figure 7, which provides an overview of the technological ecosystem supporting digital twin applications in chronic disease management.

**Figure 8:**
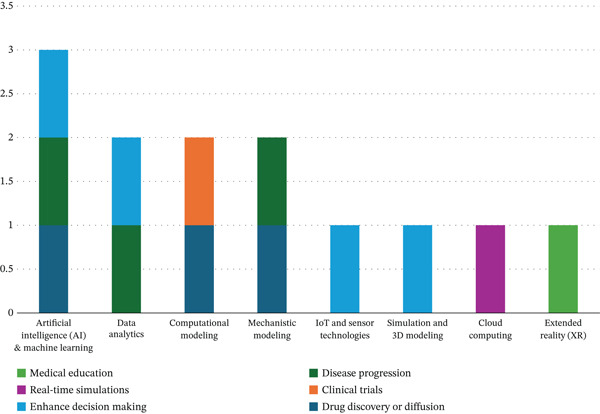
Different technologies techniques in terms of their applications.

These integrated technologies collectively improve healthcare delivery by enabling: [1] more accurate predictive capabilities, [2] personalized treatment optimization, and enhanced healthcare management efficiency. The following section systematically examines these enabling technologies as identified in the literature, detailing their specific applications and clinical significance across healthcare domains. Figure 8 illustrates the diverse technologies applied in DT systems for chronic disease management.

#### 3.5.1. AI or ML Techniques

The included studies indicate that AI and ML are among the most frequently reported enabling technologies in DT applications. These methods were used to develop predictive models, support treatment optimization, and analyze large datasets to identify latent patterns and generate clinically relevant insights. For example, AI‐based approaches were applied to identify key patient features and predict disease progression [22], while ML techniques were used for data‐driven analyses such as medical image processing [30] and sensor‐based monitoring [39]. In DT‐enabled workflows, AI/ML components can support adaptive modeling by updating predictions as new data become available. This capability is particularly relevant to personalized medicine, where DTs may be used to simulate alternative treatment scenarios and support individualized care planning [25]. In addition, AI‐driven DT approaches have been explored for early risk stratification and clinical decision support [26].

#### 3.5.2. Data Analytics and Statistical Modeling

Data analytics is a commonly reported enabling component in DT implementation. It involves analyzing large volumes of data to derive information that can support healthcare decision‐making. Across the included studies, statistical modeling approaches—including logistic regression, clustering, and related analytical methods—were used to identify patterns and relationships in patient‐level and monitoring data [22, 29]. Mancini et al. used in silico modeling combined with statistical analysis to examine viral dynamics and treatment efficacy in the context of temporary HIV treatment, aiming to help interpret inconsistent findings across clinical trial results by simulating alternative therapeutic scenarios [24]. Li et al. applied statistical modeling and data‐driven analysis to develop a DT of patients with chronic graft‐versus‐host disease (cGVHD), simulating disease progression under standard care [28]. In that study, ML‐based predictive analytics were incorporated to support individualized treatment planning within the study setting. Overall, these applications illustrate how data analytics and statistical modeling can contribute to DT development and evaluation by supporting pattern detection, prediction, and decision support.

#### 3.5.3. Computational Physiological Modeling

Computational and mechanistic modeling approaches have been widely applied in DT studies to simulate physiological processes and explore the potential effects of different interventions. These models include physics‐based representations, such as simulations of drug diffusion and transport within the body [26], as well as systems biology–based approaches, such as modeling disease‐related molecular or protein activity [33]. By simulating VP populations and estimating drug response patterns within model‐specific assumptions, DT‐based computational models may support early‐stage evaluation of therapeutic strategies and inform hypothesis generation, thereby complementing traditional experimental and clinical research rather than replacing human trials [28].

#### 3.5.4. IoT and Sensor Technologies

IoT and sensor technologies have been increasingly integrated into DT applications to support RPM and data collection. These technologies enable continuous measurement of physiological parameters—such as heart rate, blood pressure, and respiratory rate—using wearable devices and other sensors [27, 31, 39]. IoT‐enabled DT systems can collect real‐time health data and contribute to more timely assessment of a patient’s status, thereby supporting responsive clinical decision‐making.

In the included studies, textile sensor–based systems were used to detect drug‐induced changes in respiratory responses [33], and microwave‐sensing approaches were applied to monitor breathing patterns and detect falls [33]. Overall, these applications may support patient safety and continuity of care, particularly for individuals with chronic conditions who may benefit from out‐of‐clinic monitoring.

#### 3.5.5. Simulation and 3D Modeling Technologies

Simulation and 3D modeling technologies are used to create virtual representations of patients and clinical scenarios. These models can support evaluation of alternative treatment strategies, procedure planning, and workflow‐related assessments within DT‐enabled environments. For example, some included studies used 3D modeling to simulate populations of patients with hypertension to inform healthcare decision‐making and clinical trial design [26]. In addition, simulation software packages such as COMSOL Multiphysics and CST Studio Suite were used to solve physiological equations and visualize the simulated effects of interventions on human‐system behavior within the respective modeling frameworks [30]. Overall, simulation and 3D modeling tools represent key enabling technologies for developing realistic DT representations.

#### 3.5.6. Mobile Technologies

The integration of mobile technology in DTs for healthcare is demonstrated in some reviewed studies, too. Koulas I et al. [36] developed a mobile‐based scenario learning system for chronic pain self‐management, leveraging DT concepts to personalize patient education and treatment adherence. Meanwhile, Khan et al. [41] advanced RPM by implementing autonomous control in digital‐twin‐driven IoT robotic systems, where mobile platforms enable real‐time data collection and interaction between patients and their digital replicas. Together, these studies highlight mobile technology’s crucial role in enhancing DT applications—from patient education to autonomous monitoring—ultimately improving personalized healthcare delivery. The mobile components serve as both data collection hubs and interactive interfaces that bridge physical patients with their virtual counterparts.

Although simulation methods are included within the broader set of enabling technologies described in Section 3.5, they were analyzed separately because of their methodological complexity and their central role in constructing DTs [26, 29, 33–37, 41]. In contrast to AI, IoT, and data analytics—which primarily serve as supporting components within DT‐enabled systems [22, 25, 27, 31, 39]—simulation approaches (e.g., agent‐based modeling [ABM], physics‐based modeling, and virtual anatomical modeling) often constitute the computational core that drives DT model behavior and outputs [24, 26, 29, 30, 33, 38]. Accordingly, Section 3.6 treats simulation as a distinct subset within the DT technological ecosystem rather than as an independent, parallel category [24, 26, 29, 30, 33, 38].

### 3.6. Simulation Technologies in DT Applications

**Figure 9:**
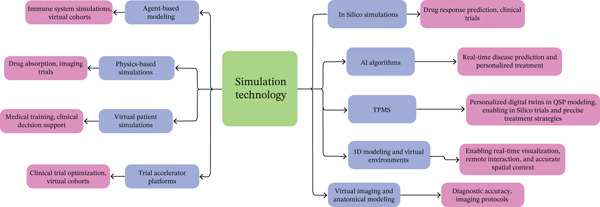
Simulation technologies in DT applications.

Simulation technologies are a core component of DT applications in healthcare. They enable the development of virtual models that simulate physiological processes, estimate patient responses to treatments, and support clinical decision‐making. A wide range of simulation methods has been applied in DT implementations, with each approach tailored to specific clinical use cases. The following section describes these simulation technologies and their applications, as summarized in Figure 9.

#### 3.6.1. ABM and In Silico Simulations

ABM is a commonly reported simulation approach in DT applications [24, 29, 38]. It enables the modeling of interactions and behaviors of individual agents within a system, where agents may represent cells, organs, or, in some cases, aggregated patient populations. In silico simulations (computer‐based models) are also frequently used—sometimes in combination with ABM—to explore drug effects, disease progression, and treatment‐response dynamics under defined model assumptions.

In the included studies, the Universal Immune System Simulator (UISS) was used as an ABM‐based platform to simulate immune system dynamics and evaluate treatment‐related effects in MS [38]. Such simulations can provide insights into disease mechanisms and support hypothesis generation for therapeutic strategies. In silico approaches have also been used to construct virtual cohorts for clinical research and trial‐related evaluations [28]. When informed by real‐world or trial‐derived data, DT‐enabled simulations may support early‐stage assessment of alternative therapeutic scenarios and complement traditional clinical studies, rather than replacing human trials.

#### 3.6.2. Physics‐Based Simulations

Physics‐based simulations use mathematical models to represent physical and physiological processes within the human body and were reported in approximately 21% of the included studies. Such approaches are important for characterizing drug absorption, distribution, metabolism, and excretion, and for estimating treatment‐related effects at the individual level.

A notable application described in the included literature involved DT development for transdermal fentanyl therapy. In these studies, DT models integrated drug uptake, PK, and PD components to estimate plasma fentanyl concentrations and support optimization of patch application timing [26, 33]. Inter‐individual variability was addressed by adjusting model parameters based on patient‐specific physiological characteristics, such as age, sex, weight, and height [26, 33].

Physics‐based simulations were also reported in virtual imaging trials. For example, one study combined computational human phantoms (XCAT) with a scanner‐specific simulator (DukeSim) to generate realistic CT images and evaluate imaging technologies and protocols under controlled conditions [30].

#### 3.6.3. AI Algorithms

AI algorithms play an important role in DT‐based healthcare applications, particularly for disease prediction and risk stratification. In the included studies by Surian et al. [22] and Chakshu and Nithiarasu [25], AI components were used to augment DT models with data‐driven capabilities, enabling the processing of complex patient data to generate clinically relevant outputs. In the context of chronic kidney disease and pneumonia‐related care, these approaches extended DT models beyond static representations toward dynamic prediction by analyzing metabolic flux patterns, simulating responses to interventions (e.g., mechanical ventilation), and supporting near real‐time assessment of patient status. Overall, integrating neural network models within DT frameworks may support personalized medicine by enabling patient‐specific virtual representations that can be updated as new data become available, with the potential to improve predictive performance and inform treatment planning [22, 25].

#### 3.6.4. VP Simulations

VP simulations are interactive, computer‐based tools that replicate clinical scenarios. They are typically designed to help healthcare professionals practice diagnostic reasoning and treatment decision‐making without the risks associated with real patient care. VP simulations can support multiple purposes, including medical training, clinical decision support, and patient education [36, 37]. In our survey, Koulas I. et al. [36] employed VPs with varying scenarios and disease stages to simulate clinical situations and gather data on how experts approach different medical conditions [36]. Additionally, these simulations offer tailored clinical guidance and feedback, enabling learners to refine their decisions based on the outcomes [37].

#### 3.6.5. Virtual Imaging and Anatomical Modeling

Virtual imaging and anatomical modeling are key components of simulation‐based approaches used in DT applications. These methods enable the creation of detailed virtual representations of human anatomy and related physiological processes, which can support the evaluation of medical devices, prediction of surgical outcomes, and analysis of operational workflows in controlled simulation settings [30]. Ho et al. used virtual imaging simulations to model airway and lung anatomy, enabling assessment of how inter‐patient anatomical variation and disease severity may influence the accuracy of airway measurements [30]. Overall, such approaches may support improvements in imaging measurement reliability and optimization of imaging protocols across clinical contexts [30].

#### 3.6.6. Trial Accelerator Platforms

Trial accelerator platforms (e.g., the Trial Accelerator DT platform) have been reported as tools for integrating and analyzing real‐world data to support clinical trial planning and evaluation [28]. These platforms can be used to construct virtual cohorts that approximate real patient populations, enabling simulation of alternative trial scenarios and estimation of expected outcomes within the assumptions of the underlying models. In this context, DT‐enabled trial accelerator approaches may complement conventional trial designs by supporting virtual cohorts and, where appropriate, synthetic or external control comparisons, potentially reducing some resource demands associated with clinical research. Such platforms may also facilitate more adaptive and patient‐informed trial design strategies, although evidence of downstream real‐world impact (e.g., time and cost savings) remains context dependent and warrants further evaluation [28].

#### 3.6.7. TPMS (Therapeutic Performance Mapping System) Technology

The TPMS has been reported as an enabling component for constructing DT‐aligned models within a QSP framework. In the study evaluating CZP treatment in moderate‐to‐severe psoriasis, TPMS was used as a systems biology–based approach that leverages ML on a human protein functional network to model drug–disease mechanisms by predicting changes in protein activity. Within that modeling context, the DT/QSP approach was used to characterize inter‐individual variability in drug response and underlying pathophysiology and to explore mechanistic subgroups and response patterns beyond dose‐related differences. Overall, this example illustrates how TPMS can contribute to DT‐enabled pharmacological modeling by supporting in silico experimentation and hypothesis generation relevant to individualized treatment strategies [34].

#### 3.6.8. 3D Modeling and VEs

3D modeling and VEs are important components in the implementation of DT systems, particularly in healthcare settings. These environments support visualization and simulation of physical systems using continuously updated data, enabling more interactive interfaces for users and clinical staff. For example, Khan et al. developed a DT‐based RPM system that used a VE to mirror the status and behavior of an autonomous robotic physical counterpart. In this configuration, the VE provided a dynamic interface through which healthcare professionals could observe and interact with the robot without being physically co‐located, supporting remote oversight and interaction [41]. Similarly, Khan et al. highlighted the use of 3D modeling to recreate care‐home environments for evaluating unobtrusive microwave sensors for DT data collection. The 3D models were used to support sensor placement and performance evaluation within a controlled, realistic setting. Overall, these studies indicate that 3D modeling and VEs can facilitate DT development and operation by providing spatial context, supporting monitoring workflows, and enabling interaction between physical and digital components [40].

Across the included studies, DT approaches generally reported encouraging technical performance, including simulation fidelity, physiologically plausible predictions within the respective modeling frameworks, and short‐term improvements in training‐related outcomes. However, only a small number of studies assessed downstream impact, such as clinical effectiveness, cost‐related outcomes, or patient‐level endpoints. Most DT implementations remained at the prototype, simulation, or feasibility evaluation stage. Overall, while DTs show promise across domains, the current evidence base suggests an early level of maturity with limited real‐world clinical validation.

## 4. Discussion

Our review suggests that the application of DT technology in clinical contexts has increased over time. Among the included peer‐reviewed studies published between 2017 and 2024, half were published in 2023–2024 [22, 23, 26, 27, 30], indicating a recent rise in publication activity. This growth may be related to broader advances in enabling capabilities, including increased computing power, improved data integration, and the maturation of AI and ML methods in healthcare settings.

Early applications identified in this review primarily focused on computational modeling and simulation to address specific clinical problems [28, 31]. For example, Gólczewski et al. developed a VP model to characterize pleural pressure changes during therapeutic thoracentesis, representing an early clinical application of DT‐related modeling [31]. During this earlier phase, DT research largely consisted of small‐scale, proof‐of‐concept studies with limited scope and validation.

The geographic concentration of DT research—particularly the higher number of studies originating from the United States—may reflect broader structural and technological factors beyond publication volume alone. Countries with strong biomedical research ecosystems, advanced computational infrastructure, and established academic–industry collaborations may be better positioned to develop and evaluate DT‐related innovations. In addition, clinical areas such as cardiology, oncology, endocrinology, and neurology appeared more frequently in the included literature, potentially because these fields often rely on quantitative biomarkers and longitudinal monitoring that are amenable to data‐driven modeling. Conversely, lower representation in other specialties may indicate barriers such as limited data availability, reduced modeling readiness, or insufficient interdisciplinary collaboration, highlighting priorities for future DT research and capacity building.

Regarding disease focus, our review indicates that DT applications have been most frequently reported in several chronic conditions, including chronic pain [25, 32, 35], diabetes [23], and chronic kidney disease [22], along with other chronic disease areas [26, 30]. This distribution is consistent with the substantial clinical burden of chronic diseases and suggests that DT approaches are being explored primarily for long‐term monitoring and management. In contrast, infectious diseases and acute conditions were less frequently addressed in the included literature [24], highlighting a potential area for future research to expand DT applications beyond chronic disease management.

The adoption of DTs appears to vary across clinical specialties. In the included literature, nephrology [22], endocrinology [23], and neurology were among the least represented specialties, whereas general medicine [26, 30, 32, 35] and medical education [28, 29] were the most frequently represented. Moderate representation was observed in oncology [27], pulmonary medicine [24, 31], and rheumatology. This distribution may reflect differences in data availability, clinical workflows, and technical readiness for DT development across disciplines.

Across publication years, the focus of DT research appears to have evolved. Applications such as RPM [33] and personalized treatment modeling [24, 32, 35] were prominent in studies published up to 2021–2022, whereas later publications increasingly described more comprehensive monitoring‐oriented frameworks. Park et al. illustrated this direction by developing a sensor‐based ML approach that uses digital biomarkers to support physical frailty assessment in older adults [33]. Studies published in 2023–2024 also showed broader application diversity and increased technical complexity, with continued emphasis on chronic disease management and integration of additional digital technologies [22, 23, 26, 30]. For example, Sai et al. proposed an AI‐enabled DT framework incorporating NFT‐related components for longitudinal monitoring, illustrating ongoing experimentation with emerging technologies within DT‐enabled care models [26].

An analysis of DT applications by target user group showed a predominance of physician‐centric tools (55%) [22, 23, 25, 27–29, 31, 32, 35], with patient‐centric solutions representing (35%) [26, 30, 33] and medical education applications comprising the remaining 5% [28, 29]. This distribution may reflect the current emphasis on clinician‐facing decision support, although the presence of patient‐oriented implementations suggests growing interest in DT‐enabled approaches for patient empowerment and self‐management.

Several key areas for the use of DTs in clinical practice were identified in our review. Personalized medicine and patient care emerged as the most frequently reported application area. DT‐based patient‐specific modeling has been used to support optimization of transdermal fentanyl dosing for chronic pain management by incorporating individual characteristics (e.g., age) into dosing‐related simulations [25, 32, 35]. Similarly, Li et al. developed a DT‐based approach to personalize standard treatment protocols for patients with chronic graft‐versus‐host disease [27].

Despite the promise of DT technologies for chronic disease management, several challenges continue to limit broader adoption. A key technical barrier is the computational complexity required to develop accurate, dynamic physiological models. High‐fidelity DTs often need to process large volumes of multimodal data (e.g., imaging, biosensor streams, and laboratory data) in near real time, which requires substantial computational resources, robust modeling frameworks, and advanced simulation capabilities [7, 9, 13]. Data integration is another major obstacle, as clinical information is frequently fragmented across electronic health record systems, medical devices, and personal monitoring tools. Limited standardization of data formats and variable data quality can hinder the creation of reliable, continuously updated patient representations [6, 9, 11].

Privacy and security also remain critical concerns. DT systems may rely on continuous flows of sensitive patient data across cloud services, IoT devices, and AI pipelines, increasing the risk of unauthorized access, security breaches, and unintended secondary use. Ensuring secure data transmission, strong encryption, and compliance with regulatory frameworks such as the General Data Protection Regulation (GDPR) and the Health Insurance Portability and Accountability Act (HIPAA) remains an ongoing requirement for safe deployment [10, 11, 13, 17].

Interoperability presents additional challenges, both between DT platforms and existing clinical information systems and across vendors’ models. The absence of shared ontologies, unified standards, and interoperable architectures can prevent seamless integration into routine clinical workflows [6, 14]. Moreover, validation and clinical trust remain key barriers: many DT models lack robust real‐world evidence, and clinicians may be hesitant to rely on simulation‐based outputs without transparent performance reporting and external validation [10, 13].

Finally, cost and infrastructure disparities—particularly in low‐ and middle‐income settings—may further constrain implementation. Developing and sustaining DT ecosystems often requires specialized expertise, reliable connectivity, sensor‐enabled environments, and access to high‐performance computing resources, which may be difficult to secure in under‐resourced health systems [14, 17]. Addressing these barriers will be essential for moving DTs beyond experimental deployments toward scalable, equitable, and clinically reliable implementation.

DT technology is increasingly being employed to enhance clinical trial efficiency and accelerate drug development processes. Coto‐Segura et al. [34] demonstrated this application through a QSP model for optimizing CZP dosing in psoriasis treatment, enabling more precise therapeutic regimens. Similarly, Mancini et al. [37] developed an in‐silico framework to analyze continuous outcomes in HIV treatment trials, showcasing how DTs can elucidate complex treatment dynamics while reducing the need for additional patient recruitment.

Given the positive benefits of vital signs monitoring sensors and speech recognition technologies, DT systems integrated with sensor networks can enable continuous patient monitoring beyond conventional clinical settings [26, 30, 33]. Khan et al. exemplified this application by developing noninvasive microwave sensor arrays for telehealth programs, showing how DT implementations could reduce hospital readmissions through automated remote monitoring systems.

Woolley et al. [42] argued that although simulations form an essential component of DTs, they are not sufficient on their own to constitute a complete DT system. The authors emphasize that the key distinction lies in the creation of a two‐way, real‐time physical‐cyber connection—a DT essentially acts as a “living simulation” that dynamically evolves alongside its physical counterpart. Such realistic simulations embedded in DTs offer significant educational value for healthcare professionals [28, 29]. In our study, we also found that Koulas et al. [34] developed a mobile scenario‐based learning platform that uses DT principles to enhance chronic pain self‐management, while Larkin et al. [37] demonstrated measurable improvements in the management of chronic hyperkalemia through VP simulations. Taken together, these cases highlight the dual potential of DTs to [1] enhance clinical education and [2] empower patient self‐management through immersive, data‐driven modeling.

A number of obstacles still stand in the way of the widespread use of DT in clinical practice, despite encouraging advancements. Numerous studies have been pointed out that there are still technical challenges in integrating data across different systems [23, 27]. The studied literature has addressed ethical issues related to patient privacy and data security with limited capacity, highlighting a need for more focus in this area. If specific measures are not taken to address infrastructural constraints, the worldwide adoption of DT technologies may potentially be constrained by resource disparities between high‐income and developing nations [26]. Standardizing evaluation standards for DT applications, improving model interoperability, and removing implementation hurdles in clinical contexts should be the main areas of future study. To show the clinical impact and cost‐effectiveness of DT technologies in various healthcare contexts and resource settings, more studies are also required.

### 4.1. Impact of Study Quality on Interpretation of Findings

The methodological quality assessment revealed substantial variability across the included studies. Empirical investigations—such as observational studies and prototype‐based evaluations—could be fully appraised using the MMAT and generally demonstrated moderate methodological rigor. In contrast, several publications proposing conceptual DT architectures or technical frameworks did not include empirical data and were therefore not eligible for MMAT assessment, in accordance with the tool’s guidelines. This heterogeneity in study design and evidentiary depth should be taken into account when interpreting the overall strength and maturity of the evidence base for DT applications in chronic disease management.

## 5. Future Research Directions

Future research on DT technologies in chronic disease management should prioritize several concrete and actionable directions to advance the field toward clinical maturity and real‐world impact.

### 5.1. Technical Development and Model Advancement

Future studies should focus on developing more robust computational frameworks capable of processing real‐time, multimodal data streams (e.g., medical imaging, IoT sensor data, and laboratory measurements). Enhancing model fidelity, reducing computational burden, and enabling adaptive or continuously updating learning mechanisms will be essential for building scalable and clinically viable DT platforms [7, 9, 13].

### 5.2. Standardization and Interoperability Infrastructure

Given the reliance of DT systems on heterogeneous data sources, future efforts should establish standardized data schemas, shared ontologies, and interoperable system architectures to facilitate seamless integration with electronic health records and medical devices [6, 9, 14]. The development of open technical standards and application programming interfaces (APIs) could substantially accelerate multi‐institutional adoption and cross‐platform compatibility.

### 5.3. Rigorous Clinical Validation Pathways

Most existing DT implementations remain at conceptual, simulation, or prototype stages. Future research should prioritize prospective clinical trials, real‐world validation studies, and benchmarking frameworks to evaluate DT accuracy, safety, and clinical utility across diverse patient populations and disease contexts [10, 13]. This includes defining standardized performance metrics, acceptable error thresholds, and transparent reporting practices.

### 5.4. Ethical, Legal, and Regulatory Frameworks

As DTs involve continuous collection and processing of sensitive patient data, future research must address ethical considerations related to long‐term monitoring, automated decision support, and data persistence. Efforts are needed to align DT applications with regulatory frameworks such as the General Data Protection Regulation (GDPR) and the Health Insurance Portability and Accountability Act (HIPAA), clarify data ownership and consent models, and establish ethical guidelines for AI‐driven clinical decision‐making [10, 11, 13, 17].

### 5.5. Data Governance and Security Architecture

Future studies should propose concrete and implementable models for secure data storage, encrypted data transmission, access control, and auditability. Research focused on identifying cybersecurity vulnerabilities and developing resilience strategies will be critical for maintaining patient trust and ensuring safe DT deployment in clinical environments [11, 17].

### 5.6. Implementation Science and Real‐World Integration

To transition DTs from experimental systems into routine clinical practice, future research should investigate implementation strategies, clinician acceptance, workflow integration, training requirements, and cost–benefit considerations. Comparative studies evaluating DT‐assisted care versus standard practice across diverse healthcare settings—including low‐resource environments—are particularly needed [14, 17].

### 5.7. Personalized and Equity‐Focused Research

Future DT research should emphasize equitable validation by assessing model performance across diverse demographic, socioeconomic, and comorbidity profiles. Additionally, studies should explore how DT technologies can actively reduce disparities in chronic disease management, rather than unintentionally exacerbating existing inequities [14].

This study encounters some limitations. We only searched for articles in English language, although study results may have been published in a language other than English. Since the results of the development of many digital systems are not published in the form of articles, many systems developed in this field may be overlooked. To solve this problem, more studies will be sought and reviewed in the future focusing on developed digital systems.

## 6. Conclusion

Our analysis indicates that DT research in chronic disease management has evolved from primarily theoretical and simulation‐based models toward more patient‐specific and clinically oriented applications. DT approaches have been explored across three main areas: personalized medicine through patient‐specific modeling, continuous or remote physiological monitoring, and AI‐enabled clinical decision support. Nevertheless, translating these technical advances into measurable real‐world benefits will require close collaboration among clinicians, computational scientists, and biomedical engineers to address ongoing challenges related to data integration, interoperability, validation, and implementation in diverse healthcare settings.

## Author Contributions

Conception and design of the study: Marsa Gholamzadeh and Amirhossein Zarei. Acquisition of data: Marsa Gholamzadeh and Amirhossein Zarei. Analysis and/or interpretation of data: Marsa Gholamzadeh and Amirhossein Zarei. Drafting the manuscript: Marsa Gholamzadeh, Amirhossein Zarei, and Fatemeh Asadi. Revising the manuscript critically for important intellectual content: Marsa Gholamzadeh and Amirhossein Zarei. Approval of the version of the manuscript to be published: Marsa Gholamzadeh, Amirhossein Zarei, and Fatemeh Asadi.

## Funding

No funding was received for this manuscript.

## Disclosure

The authors read and approved the final manuscript.

## Ethics Statement

The study involves only a review of literature without involving humans and/or animals. The authors have no ethical conflicts to disclose.

## Consent

The authors have nothing to report.

## Conflicts of Interest

The authors declare no conflicts of interest.

## Supporting Information

Additional supporting information can be found online in the Supporting Information section.

## Supporting information


**Supporting Information 1** The supporting information for this article comprise two key files: “Supplementary file A.docx”, which documents the search strategies employed across various databases along with the corresponding results.

**Supporting Information 2** PRISMA_2020_checklist DT.pdf, which contains the completed PRISMA checklist to ensure the methodology and reporting adhere to the PRISMA guidelines.

## Data Availability

Data sharing not applicable to this article as no datasets were generated or analyzed during the current study.

## Appendix A

**Table A1:** Search strategies in each database.

Database	Search strategies	Results (count of studies)
PubMed	((“chronic disease”[Title/Abstract]) OR (“chronic diseases”[Title/Abstract]) OR (“chronic illness”[Title/Abstract]) OR (“Chronic Disease”[Mesh]) OR (“chronic”[Title/Abstract])) AND ((“knowledge based”[Title/Abstract]) OR (“knowledge‐based”[Title/Abstract]) OR (“rule‐based”[Title/Abstract]) OR (“rule based”[Title/Abstract])) AND ((“Clinical Decision Support Systems”[Title/Abstract]) OR (“Decision Support System”[Title/Abstract]) OR (“Clinical Decision Support”[Title/Abstract]) OR (“electronic Clinical Decision Support System”[Title/Abstract]) OR (“CDSS”[Title/Abstract]) OR (“Decision Support Systems, Clinical”[Mesh]))	45
Scopus	(TITLE‐ABS‐KEY (“chronic disease”) OR TITLE‐ABS‐KEY(“chronic”) OR TITLE‐ABS‐KEY(“chronic illness”)) AND (TITLE‐ABS‐KEY (“knowledge based”) OR TITLE‐ABS‐KEY (“knowledge‐based”) OR TITLE‐ABS‐KEY (“rule‐based”) OR TITLE‐ABS‐KEY (“rule based”)) AND (TITLE‐ABS‐KEY (“Clinical Decision Support Systems”) OR TITLE‐ABS‐KEY (“Clinical Decision Support System”) OR TITLE‐ABS‐KEY (“Clinical Decision Support”) OR TITLE‐ABS‐KEY (“CDSS”)) AND PUBYEAR >2009	50
Web of Sciences	(TS = (“Clinical Decision Support Systems” OR “Clinical Decision Support System” OR “Decision Support System” OR “Clinical Decision Support” OR “electronic Clinical Decision Support System” OR “CDSS”)) AND (TS = (“knowledge based” OR “knowledge‐based” OR “rule‐based” OR “rule based”)) AND (TS = (“chronic disease” OR “chronic diseases” OR “chronic illness” OR “chronic”))	20
OVID	((“chronic disease”.mp. [mp = title, abstract, full text, caption text]) Or (“chronic”.mp. [mp = title, abstract, full text, caption text]) or (“chronic illness”.mp. [mp = title, abstract, full text, caption text])) AND ((“knowledge based”.mp. [mp = title, abstract, full text, caption text]) or (“Knowledge‐based”.mp. [mp = title, abstract, full text, caption text]) or (“rule‐based”.mp. [mp = title, abstract, full text, caption text]) or (“guideline‐based”.mp. [mp = title, abstract, full text, caption text])) to (yr = “2010 ‐Current” and original articles)	56

## Appendix B

**Table B1:** Application domains in terms of various disciplines.

Application domain	Studies	Median_Sample_N	With_Reported_Sample_N	Clinical_Outcomes_Yes	Conceptual_Architecture	Simulation_Only	Key takeaway (for manuscript)
Drug discovery and dosing	8	1271	6	1	0	2	In silico/QSP validation against trial/clinical data; mostly proof‐of‐concept without prospective deployment.
Medical training and education	3	32	3	0	0	0	Short‐term performance gains reported; no retention/transfer or patient outcomes.
Other/unclear	1	40606	1	0	0	0	Heterogeneous studies; reporting/validation varies.
Personalized care	3	1356	3	0	1	0	Patient‐specific modeling shows internal validity; clinical effectiveness not evaluated.
Remote monitoring	5	259	3	0	1	1	Prototype feasibility shown; small samples and limited statistical validation; no clinical endpoints.
